# Effect of Quasi-One-Dimensional Properties on Source/Drain Contacts in Vertical Nanowire Field-Effect Transistors (VNWFETs)

**DOI:** 10.3390/mi15040481

**Published:** 2024-03-30

**Authors:** Iksoo Park, Jaeyong Choi, Jungsik Kim, Byoung Don Kong, Jeong-Soo Lee

**Affiliations:** 1 Department of Electrical Engineering, Pohang University of Science and Technology (POSTECH), Pohang 37673, Republic of Korea; isparkk@postech.ac.kr (I.P.); jychoieee@postech.ac.kr (J.C.); ljs6951@postech.ac.kr (J.-S.L.); 2 Department of Electrical Engineering, Gyeongsang National University, Jinju 52828, Republic of Korea; jungsik@gnu.ac.kr

**Keywords:** quasi-one-dimensional, vertical, nanowire, field-effect transistors, source/drain, contact, quantum confinement, axial contact, radial contact

## Abstract

In this study, we investigated the influence of quasi-one-dimensional (Quasi-1D) characteristics on the source and drain contact resistances within vertical nanowire (NW) field-effect transistors (FETs) of diminutive diameter. The top contact of the NW is segregated into two distinct regions: the first encompassing the upper surface, designated as the axial contact, and the second encircling the side surface, known as the radial contact, which is formed during the top-contact metal deposition process. Quantum confinement effects, prominent within Quasi-1D NWs, exert significant constraints on radial transport, consequently inducing a noticeable impact on contact resistance. Notably, in the radial direction, electron tunneling occurs only through quantized, discrete energy levels. Conversely, along the axial direction, electron tunneling freely traverses continuous energy levels. In a meticulous numerical analysis, these disparities in transport mechanisms unveiled that NWs with diameters below 30 nm exhibit a markedly higher radial contact resistance compared to their axial counterparts. Furthermore, an increase in the overlap length (less than 5 nm) contributes to a modest reduction in radial resistance; however, it remains consistently higher than the axial contact resistance.

## 1. Introduction

The relentless miniaturization of transistors has driven the relentless pursuit of advancements to mitigate short-channel effects (SCEs) and bolster transistor performance. Strain engineering, high-κ/metal gate (HKMG) materials, and three-dimensional fin field-effect transistors (FinFETs) represent some of the key breakthroughs in this ongoing quest [1,2,3]. FinFETs, leveraging SiGe channels and extreme ultraviolet (EUV) lithography, have successfully achieved scaling down to the 5 nm regime. Gate-all-around (GAA) structures, encompassing nanowires and nanosheets, are actively being explored for technology nodes beyond 5 nm due to their superior gate controllability and enhanced current drivability characteristics [4,5,6]. Among these innovations, vertical nanowire FETs (VNWFETs) have emerged as a frontrunner for sub-3 nm technology nodes, owing to their inherent advantages in design flexibility and their ability to achieve high-density integration facilitated by the vertical channel fabrication process [7,8].

However, despite the aforementioned advantages, VNWFETs face unique challenges in contact formation.

Unlike conventional transistors, the vertical fabrication process necessitates the creation of separate contacts on both the bottom and top sides of the device. While the bottom contact analysis can use established methods, the top contact demands a more nuanced approach due to its geometrical complexity. It comprises two distinct interfaces: the circular top surface (axial contact) and the surrounding side surface (radial contact), formed during top-contact metal deposition. Addressing these contact formation challenges is paramount to propel VNWFET technology towards practical applications in next-generation electronic devices.

As the width of the nanowire (NW) shrinks, its characteristics transition towards a quasi-one-dimensional (Quasi-1D) regime. Consequently, investigating contact resistance with due consideration for NW dimensions becomes increasingly important [9,10,11,12]. Prior studies employed compact modeling to analyze parasitic components within structurally asymmetric VNWFETs, specifically focusing on 10–15 nm nanowires [13]. This modeling approach, which utilized a constant contact resistivity parameter, revealed that the top and bottom electrode asymmetry leads to imbalances in parasitic resistance and capacitance, ultimately impacting device performance. However, as NW dimensions approach the sub-10 nm scale, quantum confinement effects come into play. These effects, including bandgap widening and subband splitting, cause the nanowire’s characteristics to deviate significantly from bulk properties, profoundly influencing the contact characteristics at the metal–semiconductor interface [14].

In this study, we used a semi-classical approach that incorporates one-dimensional (1D) quantum effects specific to the top contact structure of VNWFETs. As NW dimensions decrease, the relevant density of states (DOS) exhibits a transition towards Quasi-1D characteristics, distinct from bulk behavior. This phenomenon has a significant impact on the tunneling mechanisms in both the axial and radial directions. By accounting for these quantum confinement effects, we investigated the top contact resistance along both the axial and radial directions. Additionally, we analyzed the influence of overlap distance on the contact resistance. This comprehensive examination aims to shed light on the intricate behavior of VNWFETs, a crucial step towards their continued development and practical implementation in nanoelectronic devices.

## 2. Modeling of Q1D Transport in Top Contact

**Figure 1:**
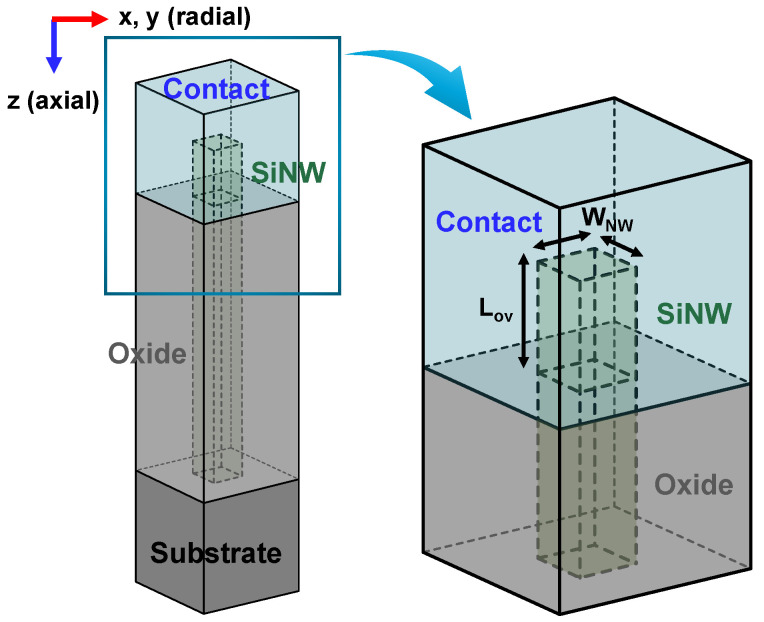
A bird’s eye view of a schematic diagram depicting a VNWFET and an enlarged view of the top contact structure of the VNWFET.

Figure 1 shows a schematic diagram of a vertical nanowire (NW) structure where the top contact comprises two interfaces: one covering the top surface, referred to as the axial contact, and the other covering the side surface with an overlap length *L_ov_*, referred to as the radial contact.

To take into account the quantum confinement effect of the Si nanowire, the quantized energy states of the NWs can be calculated by solving the Schrödinger equation to determine the discrete energy levels, as expressed by [15]:

(1) $$E_{c,n}=\frac{\hbar^{2}\pi^{2}n^{2}}{m^{*}W_{NW}^{2}},$$

where ℏ denotes the reduced Planck constant, *n* represents the quantum number for the confined direction, m^*^ is the effective mass of the electron, and *W_NW_* represents the width of the square NW. This equation provides insight into the behavior of electrons within the nanowire, crucial for understanding VNWFET characteristics and optimizing device performance for advanced electronic applications.

**Figure 2:**
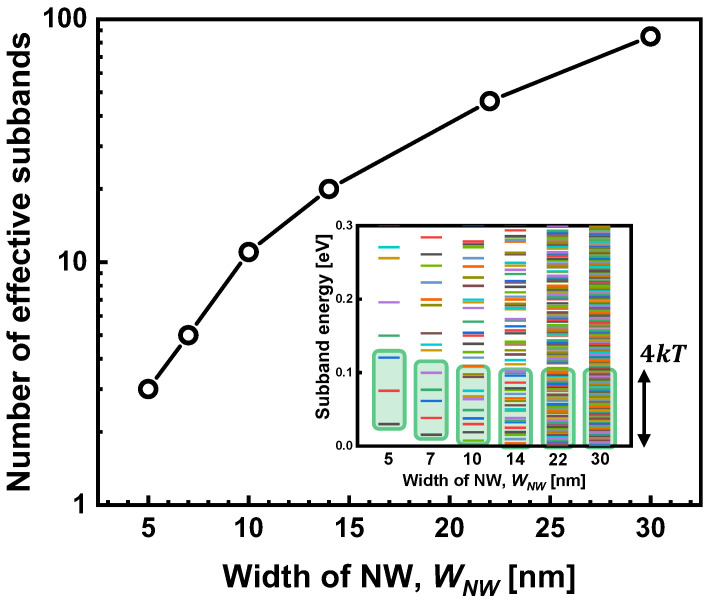
The number of effective subbands in the 4*kT* energy box from the lowest subband energy. Inset: the calculated subband energy with various widths of nanowire in the 5~30 nm range.

Figure 2 depicts the relationship between the width of the nanowire (*W_NW_*) and the number of effective subbands it possesses. These subbands are defined as the quantized energy levels within a specific range, encompassing the lowest subband energy (*E_c_*_,0_) and extending up to *E_c_*_,0_ + 4*kT*. The inset of Figure 2 provides a more detailed illustration of these quantized energy levels, calculated using Equation (1) as a function of *W_NW_*. At room temperature, the Fermi–Dirac probability function (*f(E)*) indicates that approximately 98% of the electrons populate the subbands that fall within the 4*kT* energy range. Notably, a crucial observation from Figure 2 is the significant decrease in the number of effective subbands that significantly influence electron transport. This number rapidly diminishes to 10 or fewer subbands when the WNW falls below 10 nm.

**Figure 3:**
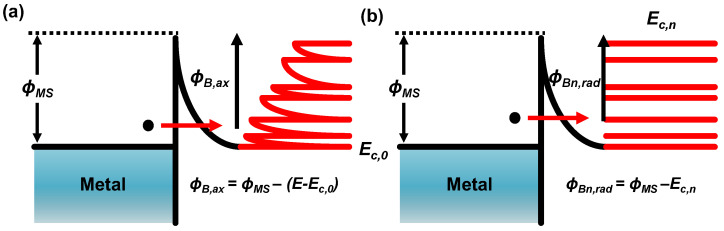
Schematic diagrams of an energy band in (**a**) the axial direction and (**b**) the radial direction with the corresponding DOS in a Quasi-1D metal–NW contact.

Continuing our analysis with reference to the energy band diagram presented in Figure 3, we can examine the mechanism of electron transport within the nanowire for both the axial and radial contacts. In both cases, electron transport occurs through a process known as field-emission tunneling. Under the assumption that the source/drain (S/D) extension regions are sufficiently doped, the metal–nanowire interface is typically modeled as an Ohmic-like Schottky contact. This assumption is based on the nanowire possessing a sufficiently small width and a doping concentration of 1 × 10^20^ cm^−3^. However, a critical distinction between the axial and radial contacts arises due to the influence of a parameter stemming from the inherent Quasi-1D quantum effects.

The density of states (DOS) in the axial direction of the nanowire of each subband energy can be calculated using the following equation [12]:

(2) $$g\left(E\right)=\frac{1}{\pi \hbar }\sqrt{\frac{m^{*}}{2\left(E-E_{c,n}\right)}},$$

where *E* is the energy of the electron, and *E_c,n_* are the quantized subband levels. In an NW with a sub-nanometer diameter and significant length, carrier motion is confined to a 1D space. Consequently, carriers traverse a continuous spectrum of energy states along the wire’s axis, while their motion in the radial direction is constrained to specific quantized energy levels. In the axial contact, tunneled electrons encounter a continuum of energy levels, whereas along the radial direction, they encounter discrete energy levels. The tunneling probability was calculated using the Wentzel–Kramers–Brillouin (WKB) approximation method, which is expressed as

(3) $$T\left(E\right)=exp\left(-\frac{8}{3\hbar }\sqrt{\frac{\epsilon_{s}m^{*}}{qN_{d}}}\left(\phi_{B}-V\right)\right),$$

where *ϵ_s_* is the permittivity of the semiconductor, *N_d_* is the doping concentration of the semiconductor, *ϕ_B_* is the tunneling barrier height, and *V* is the bias voltage.

The tunneling thickness was determined by calculating the depletion width of the metal–semiconductor contact using the formula $W_{d}=\surd ((2\epsilon_{s}(\phi_{B}-V))/({qN}_{d}))$, where Fermi-level pinning effects were disregarded due to the specific charge characteristics of one-dimensional nanowires [16]. The tunneling barrier height (*ϕ_B_*) for both the axial and radial contacts was obtained using Equations (4) and (5), respectively. As shown in Figure 3, electrons encounter distinct barrier heights depending on their transport directions. Those tunneling through the axial direction face a continuum of energy states, suggesting that the axial barrier height is continuous, given by

(4) $$\phi_{B,ax}=\phi_{MS}-\left(E-E_{c,0}\right)$$

Those traversing the radial direction face the quantized barrier heights that depend on the quantized levels:

(5) $$\phi_{B_{n,rad}}=\phi_{MS}-E_{c,n}$$

For the metal contacts, titanium was selected, commonly employed as the initial layer in modern CMOS processes, setting the metal–semiconductor work function difference ($\phi_{MS}$*)* to 0.3 eV in this scenario.

## 3. Calculation of Axial and Radial Contact Resistances

**Figure 4:**
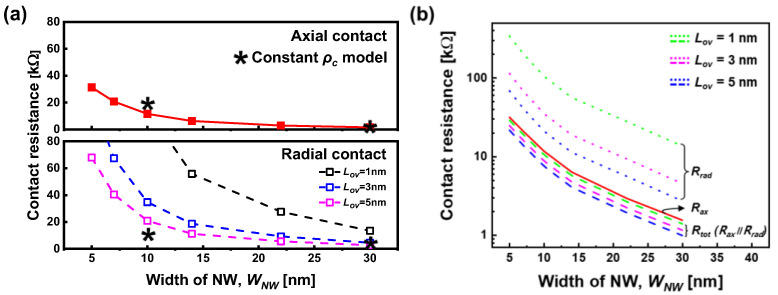
(**a**) Calculated contact resistance of axial (*R_ax_*) and radial (*R_rad_*) contacts with varying *W_NW_* and *L_ov_*. The asterisks (*) denote values calculated using the constant *ρ_c_* model [13] and (**b**) *R_ax_* (solid), *R_rad_* (dot), and *R_tot_* (=*R_ax_∥R_rad_*) (dash) with varying *W_NW_* and overlap lengths (*L_ov_* = 1, 3, and 5 nm).

Figure 4a shows the contact resistance of the axial (*R_ax_*) and radial (*R_rad_*) contacts, determined from the current density calculated using the following equation:

(6) $$J_{S\to M}=qv\int_{E_{c,0}}^{E_{c,0}+4kT}g\left(E\right)f\left(E\right)T\left(E\right)dE,$$

where *q* is the electron charge, *v* is the velocity of the electron, *k* is the Boltzmann constant, *T* is the temperature, *g(E)* is the density of states, *f(E)* is the Fermi–Dirac distribution probability, and *T(E)* is the tunneling probability.

In the case of a 30 nm nanowire width (*W_NW_*), our proposed approach yields results for the axial contact resistance (*R_ax_*) and radial contact resistance (*R_rad_*) that are comparable to those obtained using the previously established constant contact resistivity (*ρ_c_*) model, which uses experimentally determined contact resistivity values [13]. This suggests that for larger-diameter NWs, the *ρ_c_* model can provide a reasonable approximation. However, the *ρ_c_* model suggests that the radial resistance (*R_rad_*) can be lowered compared to the axial resistance (*R_ax_*) by simply adjusting the overlap length (*L_ov_*). For instance, with an overlap length of 5 nm and *W_NW_* of 10 nm, the *ρ_c_* model predicts an axial resistance of approximately 20 Ω and a radial resistance of approximately 10 Ω. This implies that the total contact resistance can be primarily attributed to the combined effects of both axial and radial resistances. However, for NW diameters below 15 nm, a significant discrepancy emerges between the two models. As previously mentioned, only quantized energy levels are relevant for electron transport in the radial direction. Consequently, the influence of *W_NW_* on radial resistance becomes considerably more pronounced compared to in the axial direction, highlighting the limitations of the ρc model for sub-15 nm NWs. Consequently, even with an augmented *L_ov_*, *R_rad_* consistently maintains higher values than *R_ax_*.

In Figure 4b, the influence of *R_ax_* and *R_rad_* on the total resistance of the top contact, *R_tot_*, is shown. According to our proposed model, due to the quantum confinement effect, *R_rad_* consistently exhibits a larger value compared to *R_ax_* regardless of *L_ov_*. Therefore, *R_tot_* can be represented as the parallel of *R_ax_* and *R_rad_*, confirming that it is primarily determined by *R_ax_*.

## 4. Conclusions

This investigation explores the impact of contact structure on the electrical characteristics of quasi-one-dimensional vertical nanowire field-effect transistors (VNWFETs). These devices feature a distinctive top contact, consisting of two regions: the upper circular area and the circumferential contact encircling the sidewall. Due to the inherent one-dimensional nature of these contact interfaces, they exhibit distinct contact resistance behaviors. The conventional method of calculating contact resistance, based on contact area and specific contact resistivity between the metal and semiconductor, proves inadequate for nanowire contacts with sub-nanometer diameters due to quantum confinement effects. In Quasi-1D VNWFETs, electrons traveling in the radially confined direction encounter strictly quantized energy levels, greatly limiting their tunneling probability compared to the continuous energy states available along the axial direction. Consequently, as nanowire dimensions decrease, electron tunneling in the radial direction experiences a significant reduction in current compared to tunneling along the axial direction. This critical observation underscores the necessity of considering both distinct interface types for effective device performance optimization. Our findings emphasize the crucial need for accurate modeling of quantum confinement effects, particularly in VNWFETs approaching or falling below the 10 nm threshold.

## Data Availability

The data presented in this study are available on request from the corresponding author. The data are not publicly available due to confidentiality request.
